# Synthetic Studies on Tetracyclic Diquinane Lycopodium Alkaloids Magellanine, Magellaninone and Paniculatine

**DOI:** 10.3390/molecules28031501

**Published:** 2023-02-03

**Authors:** Takeru Saito, John Mark Awad, Wei Zhang

**Affiliations:** Department of Chemistry and Center for Green Chemistry, University of Massachusetts, Boston, MA 02125, USA

**Keywords:** lycopodium alkaloid, total synthesis, magellanine, magellaninone, paniculatine

## Abstract

(–)-Magellanine, (+)-magellaninone, and (+)-paniculatine are three natural products isolated from the *Lycopodium* family that share a unique 6-5-5-6-fused tetracyclic diquinane core skeleton. Several members of this family have potent s anti-inflammatory and acetylcholinesterase-inhibitory properties and are under development for the treatment of Alzheimer’s and other neurodegenerative diseases. Several research groups have undertaken the formal and total syntheses of this class of natural products. This review highlights over 20 reported total syntheses of these three alkaloids and the development of synthetic methods for the assembly of their core skeletons.

## 1. Introduction

**Figure 1:**
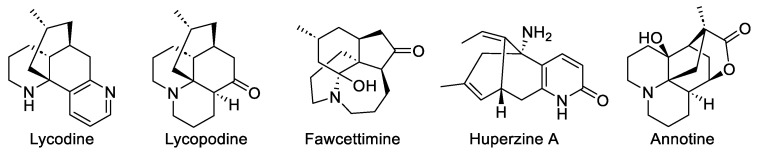
Structures of lycodine, lycopodine, fawcettimine, huperzine A, and annotine.

The lycopodium alkaloids, isolated from the club moss Lycopodium and named by Linnaeus in 1753, represent a class of several-hundred natural products that were divided by Backer into the following four sub-families based on their secondary metabolites [1]: lycodine, lycopodine, fawcettimine, and miscellaneous (such as huperzine A and annotine) (Figure 1). They have attractive biological and chemical properties. Some members of this family display potent inhibitory activity against acetylcholinesterase, which is the key brain enzyme responsible for the rapid degradation of the neurotransmitter acetylcholine [2,3]. This inhibition delays the hydrolysis of acetylcholine, thus increasing its levels in the synaptic cleft. Therefore, many members of the lycopodium family have been explored for the treatment of Alzheimer’s and other neurodegenerative diseases [1]. 

The literature suggests that huperzine A (lycodine-type), annotine (miscellaneous-type), and related compounds could be used for the treatment of inflammatory diseases due to their ability to induce anti-inflammatory effects through the mitigation of cytokine expression. Huperzine A inhibits cytoplasmic IκBα degradation and nuclear factor-KB (NF-KB) translocation, while annotine multifunctionally decreases IL-2 and IL-6 cytokine expression in dendritic cells, and, conversely, increases IL-10 secretion and promotes T-cell maturation directed toward a Th2/Treg phenotype [4,5]. Similar anti-inflammatory effects ameliorate the pathogenesis of experimental autoimmune encephalomyelitis by decreasing inflammatory cell infiltration in the spinal cord through the inhibition of chemokine ligand 2 production [6]. Furthermore, studies show that lycopodium alkaloids confer an significant inherent inhibitory effect against foam cell formation, a characteristic of atherosclerotic lesions [7]. 

**Figure 2:**
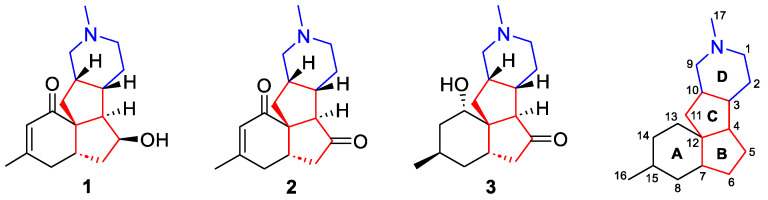
Structures of (–)-magellanine (**1**), (+)-magellaninone (**2**), and (+)-paniculatine (**3**), and carbon numbering of the core.

The structures of (–)-magellanine (**1**) and (+)-magellaninone (**2**) of the fawcettimine class, along with (+)-paniculatine (**3**) of the lycopodine class, are shown in Figure 2. These three alkaloids were isolated from *Austrolycopodium magellanicum* and *Austrolycopodium paniculatum* by Castillo and coworkers in the 1970s [8,9,10,11]. Structurally, compounds **1**–**3** share a 6-5-5-6-tetracyclic framework (ABCD), with a diquinane core (in red) and an *N*-methyl piperidine ring (in blue). Magellanine and magellaninone share the ACD-ring but vary in the oxidation state at C5. Paniculatine (**3**), on the other hand, comprises a unique AB-ring with the same C5 as in **2**. However, the A-ring consists of a 1,3-hydroxymethyl substitution in a *trans*-relationship, thus differing from the enone found in **1** and **2**.

To the best of our knowledge, no significant biological studies have been conducted on **1–3**. However, several synthetic reports suggest that due to the structural similarities between these three compounds and others in the lycopodium family, **c** could also possess strong inhibitory activity against acetylcholinesterase, if not against other targets.

In this review, we wish to describe several reported syntheses of **1**–**3** through an initial introduction of each report’s retrosynthesis, followed by a description of the synthetic route designed for the construction of these complex natural products. Attention is paid to the key steps of the total synthesis. In addition, we also briefly highlight several other notable syntheses for the construction of the ring systems. 

## 2. Early Synthetic Efforts from 1986–1993

Before describing the reported total syntheses of (–)-magellanine, (+)-magellaninone, and (+)-paniculatine (**1**–**3**), it is worth highlighting some of the efforts before Overman’s 1993 landmark total synthesis due to their unique strategies in the context of constructing the tetracyclic framework that defines these diquinane alkaloids. Of course, several other synthetic studies have been reported throughout the years between 1993 and the present [12,13,14], some of which will be briefly discussed in Section 4. In lieu of traditional retrosynthetic analyses for these syntheses described in Section 2, a condensed scheme will be shown to highlight the key transformations utilized in these strategies.

### 2.1. Paquette’s 1986 Synthesis

**Scheme 1:**
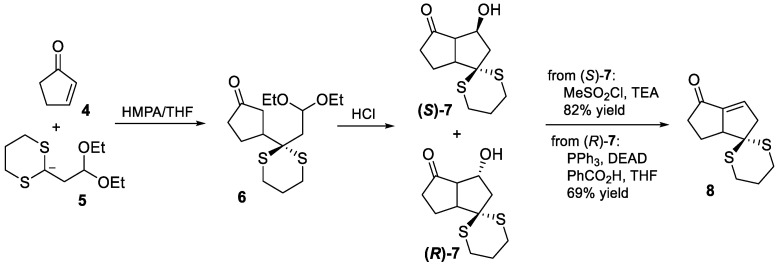
Paquette’s 1986 synthesis. HMPA = hexamethylphosphoramide. TEA = triethylamine. DEAD = diethyl azodicarboxylate. THF = tetrahydrofuran.

In 1986, Paquette and coworkers first reported a synthetic method to construct the central 5-5-fused bicyclic core, fitted with appropriate functionalities that would later be elaborated (Scheme 1) [15]. The model study was initiated by a 1,4-addition on cyclopentenone **4** with 1,3-dithiane carbanion **5**, liberated from its sodium salt [16]. A similar chemistry had been developed by Heathcock and coworkers [17], wherein they utilized the ketal for conjugate addition. Via the treatment of **6** with dilute HCl, a mixture of four or more products emerged; the two of interest are shown above. For the two ß-hydroxy-ketone enantiomers of **7**, unique conditions were applied for their elimination to form **8**. For the (*S*)-enantiomer, a simple conversion of the hydroxy group to its mesylate followed by elimination with triethylamine afforded **8** at an 82% yield. On the other hand, the (*R*)-enantiomer underwent elimination through the Mitsunobu conditions [18], with PPh_3_ and DEAD, to afford **8** in 69% yield. This sequence results in pseudo-bicyclic pentanone **8** in a 32-38% yield over the three steps. However, one drawback they had encountered was that the α,ß-unsaturation in the indicated position is unfavored due to ring strain, similar to that observed by Agosta and Wolff [19]. Via treatment with DBN, Paquette and coworkers found that the double bond readily isomerizes to the *ß,γ*-position. This posed a hurdle that would later be tackled in their 1993/94 synthesis, which is described in this review; the enone functionality is pivotal to efficiently constructing the rest of molecules **1** and **2**. 

### 2.2. Overman’s 1989 Synthesis

**Scheme 2:**
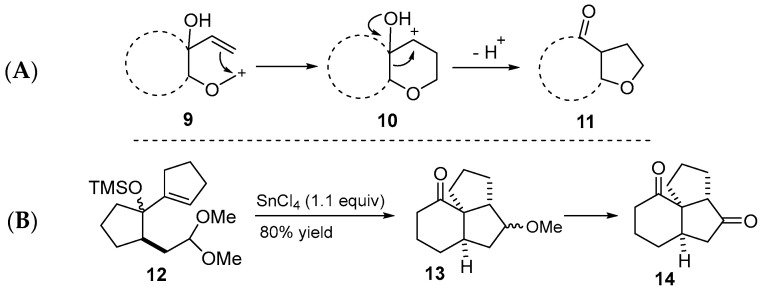
(**A**) Prins-pinacol rearrangement. (**B**) Overman’s 1989 strategy. TMS = trimethylsilyl.

In 1989, Overman and coworkers, on the basis of their previous work involving a cationic cyclization-pinacol rearrangement that was undertaken to construct oxygen heterocycles [20,21], reported a “ring-enlarging cyclopentane annulation” sequence to construct the 6-5-5-fused tricyclic ring system **14** found in **1**–**3** (Scheme 2A) [22]. This unique transformation is initiated by 1,3-proton transfer, shown in **9**, which subsequently undergoes pinacol rearrangement (through intermediate **10**) to form the exo-aldo-tetrahydrofuran ring of **11**. Dimethyl-acetal **12** exploits this chemistry to stereoselectively construct the 6-5-5-tricyclic core at an 80% yield over one step (Scheme 2B). The pinacol rearrangement builds the northern cyclopentane ring as a single diastereomer, while the stereochemistry of the methoxy group is influenced by the geometry of the silyl enol ether. The use of the ß-isomer yields the α-methoxy as a single enantiomer, while the use of the α-silane results in a 2:1 mixture of the α- and ß-appendages. Further oxidation is also exemplified to form diketone **14**. Similar to Paquette’s report, Overman and coworkers continued their synthesis, which is examined later in this review, to achieve the first total synthesis of **1** and **2**. 

### 2.3. Mehta’s 1987 and 1990 Syntheses

**Scheme 3:**
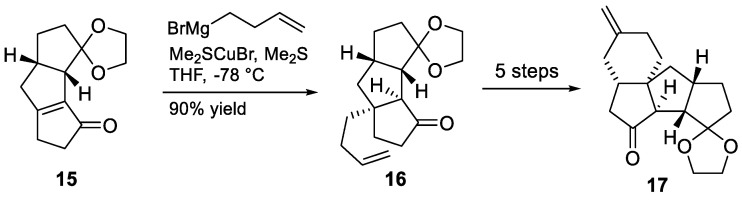
Mehta’s 1987 synthesis. THF = tetrahydrofuran.

In 1987 and again in 1990, Mehta and coworkers reported their synthetic efforts towards the construction of the carbon framework of **1**–**3**. For their 1987 strategy [23], readily accessible acetal **15** was alkylated via conjugate addition with an alkyl Grignard reagent (Scheme 3). Consequently, a series of five steps was employed to construct the pseudo-tetracyclic scaffold of **17**. First, **16** was converted to its corresponding enone via a selenylation-selenoxide elimination sequence with the unsaturation resulting in the southernmost C-C bond of the cyclopentanone ring. Wacker oxidation of the terminal olefin to the methyl ketone was performed using Tsuji conditions [24], after which exposure to basic conditions gave rise to an intramolecular Michael addition to construct the six-membered ring. The resulting ketone was selectively capped through the Wittig conditions to afford exo-methylene **17**. This is a key handle that they envisioned would be required for the synthesis of **1**–**3**. 

**Scheme 4:**
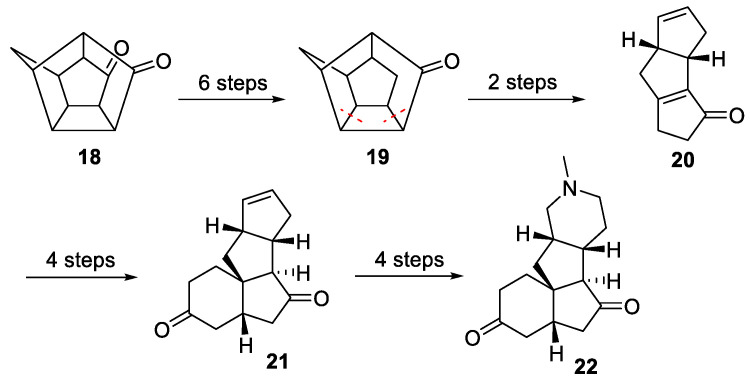
Mehta’s 1990 synthesis.

Several years later, Mehta’s group developed a new approach that used a highly constrained pentacyclic dione to construct the 5-5-5-fused tricyclic intermediate **20** as their key molecule to build the core tetracyclic scaffold (Scheme 4) [25]. First, over six steps, dione **18** was converted to ketone **19**, which was then subjected to a thermally induced [2+2]-cycloreversion reaction under flash vacuum pyrolysis and a transposition of the enone to reveal **20** [26]. Over the course of the next four steps, the southern portion of **20** was elaborated to construct the cyclohexanone moiety in **21**, which, after another four steps, resulted in diketone **22**, with the methyl-piperidine incorporated. The construction of the cyclohexanone ring is fairly similar to the aforementioned 1987 strategy, but differs at the last stage, wherein the ketone was kept intact as opposed to the Wittig route involving the capping of the ketone. The stereochemistry of **21** follows from the established literature that bicyclo[3.3.0]octanes predominantly react on the convex face, driven by the pseudo-axial proton, which is vicinal to the all-carbon quaternary center. With **22** in hand, Mehta and coworkers envisioned the completion of **1**–**3**, albeit with no reported success (to the best our knowledge). 

### 2.4. Crimmins’ 1993 Synthesis

**Scheme 5:**
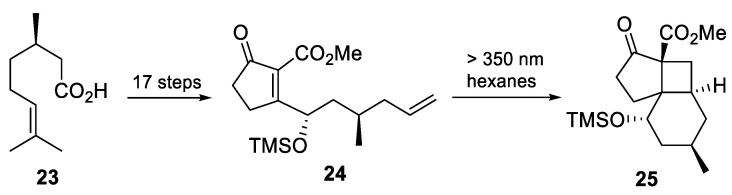
Crimmins’s 1993 synthesis. TMS = trimethylsilyl.

In 1993, Crimmins and coworkers reported a cycloaddition strategy as a potential precursor for the complete or partial construction of the tetracyclic scaffold of **1**–**3** (Scheme 5) [27]. In their approach, carboxylic acid **23** was first transformed in a lengthy but stereoselective 17-step synthesis to obtain **24**. Consequently, a [2+2]-photoinduced cycloaddition took place with irradiation in the near-UV range to afford **25** [28]. This intramolecular cycloaddition is the first example of its kind that demonstrates asymmetric induction directed by a stereocenter on a four-carbon tether [29,30]. With four stereocenters, there are 16 conformations (eight chair and eight boat) in which the substrates can interact. The anti-intermediate is primarily in a boat conformation, which places the hydroxyl and methyl groups in the energetically favorable equatorial positions. However, the *syn*-photosubstrate reacts in a chair confirmation, which puts the hydroxyl and methyl groups in the axial positions. This gives rise to the mixture of diastereomers found in this reaction, centered around the 4-6-fused ring system. Similar to Mehta, Crimmins and coworkers envisioned that this strategy could be applied to the total synthesis of **1**–**3**, but no reports have been published to the best of our knowledge. 

## 3. Total Synthesis of Magellanine, Magellaninone, and Paniculatine (1–3)

This section describes the total syntheses of **1**–**3**. Ten research groups’ studies reported between 1993 and 2022 are highlighted. 

### 3.1. Overman’s 1993 Synthesis of (–)-Magellanine and (+)-Magellaninone

**Scheme 6:**
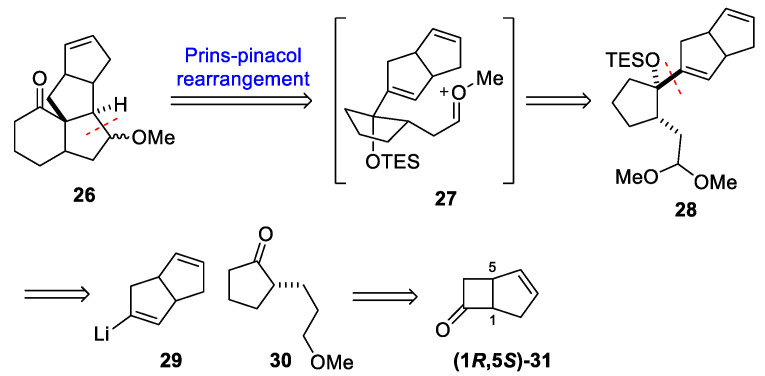
Overman’s 1993 retrosynthesis. TES = trimethylsilyl.

Four years after their initial report, Overman and coworkers reported the first asymmetric total synthesis of (–)-magellanine (**1**) and (+)-magellaninone (**2**) in 25 and 26 steps, respectively [31]. As seen in the retrosynthesis (Scheme 6), they envisioned that the tetracyclic skeleton of **1** and **2**, outlined in **26**, could be constructed by their previously developed Prins-pinacol rearrangement of acetal **28** through oxonium intermediate **27**. This transformation was to be stereocontrolled so that Prins cyclization would occur on the convex face of the cis-fused bicycle **27**, as illustrated. It was thought that **28** was constructed from the 1,2-addition of bicyclic vinyllithium **29** onto ketone **30**; the latter traced back to an enantiopure bicyclo[3.2.0]heptenone substrate **31**. 

**Scheme 7:**
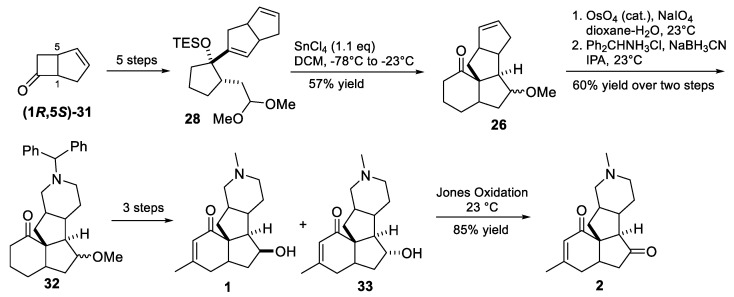
Overman’s synthesis. TES = triethylsilyl. DCM = dichloromethane. Jones oxidation = CrO_3_, H^+^.

The synthesis commenced with the preparation of dimethylacetal **28** through five steps (Scheme 7). This sequence, while not shown, includes a regioselective ring expansion of (+)-**31**, as described by Cohen [32,33], and the conversion of the resulting *α*-methylthioketone to a vinyl triflate, which was then converted to vinyl iodide with *N*-iodosuccinimide [34]. The iodide was lithiated and then added to cyclopentanone **30** (vide supra) which was subsequently transformed to **28** through standard functional group manipulation protocols. 

With enantiopure **28** in hand, the key Prins-pinacol rearrangement was executed. This rearrangement occurs via the acid-catalyzed demethoxylation of acetal to form a methoxycation, which then undergoes cyclization with the proximal olefin to complete the Prins reaction. In the presence of an allylic silyl-ether, the Prins reaction can cascade into a Pinacol rearrangement to eliminate the silyl group and form a ketone. The beauty of this transformation is the establishment of five of the six stereocenters found in **1** and **2** with complete stereocontrol throughout the sequence.

With the tetracyclic 6-5-5-5-fused skeleton constructed, the last major hurdle was the construction of the piperidine from the cyclopentene ring. This was performed via the oxidative cleavage of the double bond with osmium tetroxide [35], followed by double reductive amination of the resulting di-aldehyde with a primary amine to afford **32** [36]. To complete the total synthesis of **1** and **2**, intermediate **32** was first transformed over three steps to achieve **1**, namely, through a Saegusa–Ito oxidation to install the *α,ß*-unsaturated ketone [37]. Through this sequence, they were able obtain a separable mixture of **1** and **33** as C5-epimers, which, fortunately, could be oxidized with Jones conditions to afford **2** in one step. 

### 3.2. Paquette’s 1993/1994 Synthesis of (–)-Magellanine and (+)-Magellaninone

**Scheme 8:**
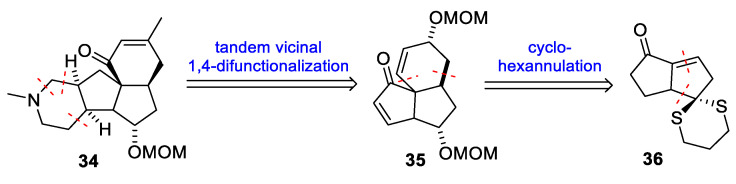
Paquette’s retrosynthesis. MOM = methoxymethyl.

First in 1993 and later in 1994, Paquette and coworkers reported the total synthesis of **1** and **2** through a three-fold annulation strategy [38,39]. By examining the retrosynthesis (Scheme 8), tetracyclic precursor **34** could be derived from **35** through a tandem vicinal 1,4-difunctionalization annulation initiated by a nitrogen-containing nucleophile [40]. The six-membered ring in **35** was thought to be constructed from **36** through a dual Michael ring annulation, encapsulating the three-fold annulation strategy. This was the method of choice, as opposed to a more conventional Diels–Alder sequence, to obtain the correct regioselectivity of the double bond and the oxygenated carbon atom. This retrosynthesis scheme takes advantage of a reliable method to control stereochemistry at each step, with the safety net of a kinetic resolution to resolve a mixture of enantiomers.

**Scheme 9:**
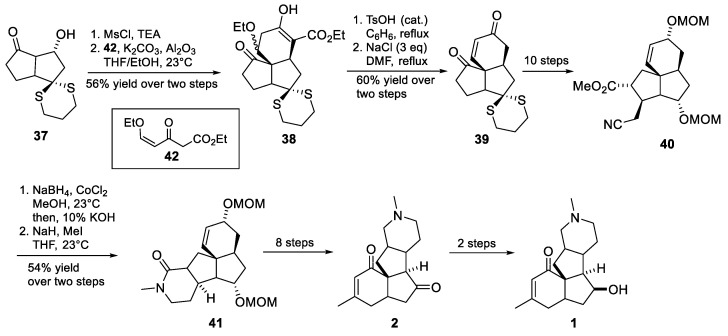
Paquette’s 1993/94 synthesis. MsCl = mesyl = methanesulfonyl chloride. TEA = triethylamine. THF = tetrahydrofuran. TsOH = tosylic = *p*-toluenesulfonic acid. DMF = *N*,*N*-dimethylformamide. MOM = methoxymethyl.

To commence its synthesis (Scheme 9), *ß*-hydroxy ketone **37** was first mesylated and eliminated to form the α,ß-unsaturated enone, to which α,ß-unsaturated keto-ester **42** was added under basic conditions to afford pseudo-tricyclic **38** [41,42]. The stereochemistry of the annulation results from two main factors: the presence of the *ß*-axial tertiary proton in the diquinane scaffold that makes the same (bottom) face sterically unfavorable, and the strong thermodynamic favorability of the 5-5-fused ring system towards being cis- rather than trans-fused [43,44]. Thus, **42** is added onto the top face, which produces the shown desired configuration. Enol **38** was then subjected to acidic conditions to promote ß-elimination. This was followed by a Krapcho decarboxylation to yield enedione **39** [45]. 

With a reliable method to construct the pseudo-tricyclic scaffold with the proper functionalities in place, a ten-step sequence was performed to construct tricyclic **40**. While not shown, this tedious stepwise process included: the conversion of a cyclohexanone moiety to an *α,ß*-unsaturated, MOM-protected hydroxy group; the conversion of the 1,3-dithiane to a MOM-protected alcohol; and then the careful transformation of the cyclopentenone ring to a 2-ethylnitrile methylester, resulting in intermediate **40**. With **40** constructed, the piperidone ring was constructed through an established protocol to afford **41**. The use of NaBH_4_ and Co(Cl)_2_ selectively reduces nitriles to their corresponding primary amines [46,47,48], which were subjected to basic conditions to form the lactam and, subsequently, treated with methyliodide to form the signature methylamide found in **1** and **2**. 

To complete the synthesis of **1**, an eight-step sequence was performed. Among others, a notable transformation was the conversion of the protected α,ß-unsaturated alcohol to an ß-methyl enone, taking note of the new connectivity of the oxygen atoms. This allylic rearrangement was performed to shift a secondary alcohol two carbons over, which was then oxidized to form the ketone via Jones oxidation. With enedione **2** in hand, two steps were performed to obtain **1**, with one such step consisting of a hydride reduction, which inadvertently formed the incorrect hydroxy stereocenter; however, conventional Mitsunobu displacement and elimination resulted in epimerization [49].

### 3.3. Sha’s 1999 Synthesis of (+)-Paniculatine

**Scheme 10:**
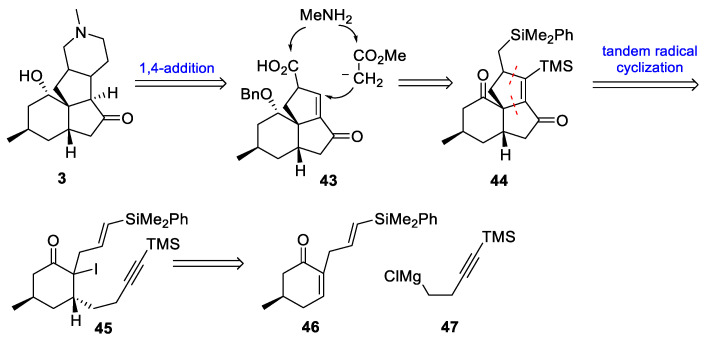
Sha’s retrosynthesis. Bn = benzyl. TMS = trimethylsilyl.

In 1999, the third member of this sub-family of *Lycopodium* alkaloids, (+)-paniculatine, was synthesized by Sha and coworkers for the first time [50]. This builds off their previous work involving the utilization of α-carbonyl radical cyclization reactions to efficiently construct complex natural products [51,52,53]. In their retrosynthetic analysis, they believed that the piperidine ring of **3** could be constructed from a 1,4-addition of an acetate fragment, followed by nitrogen insertion, utilizing methylamine as a linchpin (Scheme 10). Furthermore, tricyclic enone **43** was thought to be accessible from enedione **44**, itself being traced back to **45** via a tandem radical cyclization, to close the 5-5-fused diquinane core. Iodide **45** could be simply stitched together with 1,4-addition of Grignard reagent **47** onto enone **46** [54]. 

**Scheme 11:**
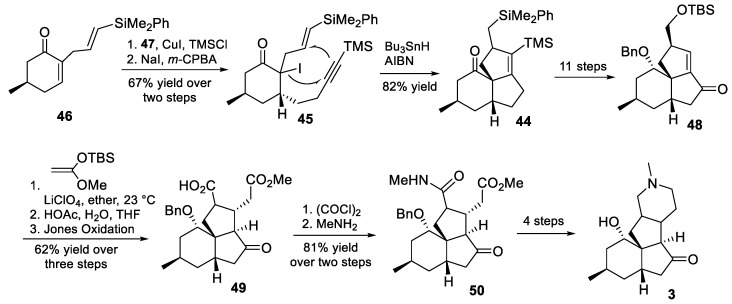
Sha’s 1999 Synthesis. TMSCl = trimethylsilyl chloride. AIBN = azobisisobutyronitrile. TMS = trimethylsilyl. Bn = benzyl. TBS = *t*-butyldimethylsilyl. THF = tetrahydrofuran. Jones oxidation = CrO_3_, H^+^.

To commence their synthesis, Sha and coworkers constructed iodide **45** from **46** through the following procedure: Cu(I)-mediated 1,4-addition of **47** onto **46**, followed by trapping with TMSCl, and iodination with NaI/*m*-CPBA to yield **45** at a 67% yield over two steps [55,56] (Scheme 11). This set the stage for their previously developed and optimized tandem radical cyclization with Bu_3_SnH and AIBN to yield tricyclic intermediate **44** at an 82% yield with the correct stereochemistry. With enedione **44** in hand, a series of 11 steps was utilized to construct enone **48** with a *γ*-TBS-silyl enol ether. Then, a five-step sequence occurred to yield **50**. First, the TBS-methyl ketene acetal underwent a stereoselective Mukaiyama–Michael addition to enone **48** [57], followed by the removal of the TBS group with acetic acid, and lastly Jones oxidation of the resulting primary alcohol to the corresponding carboxylic acid. As expected, the 1,4-addition occurred on the less-hindered face, which led to the construction of a carboxylic acid intermediate (not shown here) at a 62% yield over three steps in the correct stereochemistry. From there, the acylation of carboxylic acid **49** with oxalyl chloride and methylamine yielded amide **50**, which was then transformed through four steps to yield **3** in 23 longest linear steps.

### 3.4. Liao’s 2002 Synthesis of (±)-Magellanine

**Scheme 12:**
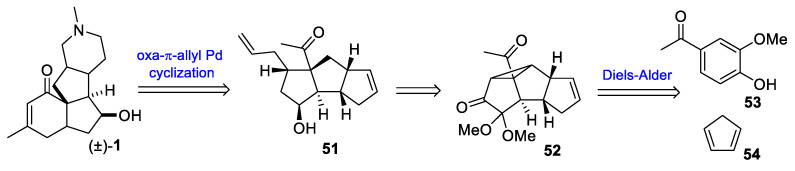
Liao ’s retrosynthesis in 2002.

In 2002, Liao and coworkers disclosed a total synthesis of **1** as a racemic mixture [58]. As there was no graphically depicted retrosynthesis in the original paper, we have taken the liberty to draw a brief scheme to explain their thought process (Scheme 12). First, **1** was thought to be traced back to ketone **51** through an intramolecular cyclization with oxa-π-allyl Pd(II) intermediates and an oxidative cleavage/reductive amination sequence to construct the piperidine core. Tricyclic **51** could be constructed from tetracyclic intermediate **52**, which, in turn, originated from the manipulation of a Diels–Alder adduct formed by **53** and **54.**

**Scheme 13:**
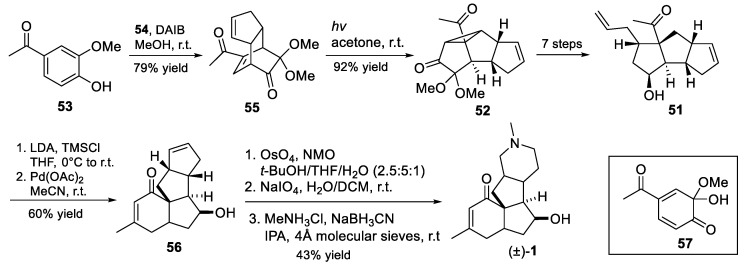
Liao’s racemic synthesis of **1**. DAIB = diacetoxyiodobenzene. LDA = lithium diisopropylamide. TMSCl = trimethylsilyl chloride. THF = tetrahydrofuran. NMO = *N*-morpholine *N*-oxide. DCM = dichloromethane. IPA = *i*-propyl alcohol.

After a thorough investigation of the conditions from their previous report [59], Liao and coworkers set off to initiate a Diels–Alder-ODPM (oxa-di-π-methane) rearrangement cascade reaction [60,61], which occurs through intermediate **57** (Scheme 13). This cascade exploits the innate nature of each chemical reaction to stereoselectively construct the four contiguous stereocenters at an early stage, whose result is intermediate **52**. In addition, the careful placement of an electron-withdrawing acetyl group further supplements this transformation [62]. From there, a seven-step sequence was utilized to open the cyclopropane ring, stereoselectively form the secondary alcohol, and install an allyl side. With intermediate **51** in hand, an intramolecular cyclization with oxa-π-allyl-Pd(II) intermediates took place to afford tetracyclic enone **56** at a 60% yield over three steps. 

With the ABC-ring system in place, the last piece of the puzzle was to form the D ring. This was performed through oxidative cleavage with osmium tetroxide to cleave the cyclopentene ring, and then double reductive amination to construct the piperidine ring [36,63,64]. This racemic synthesis of **1** was completed in 14 steps at a 9% yield, thus constituting a significant improvement to its predecessors. 

### 3.5. Ishizaki’s 2005 Synthesis of (±)-Magellanine 

**Scheme 14:**
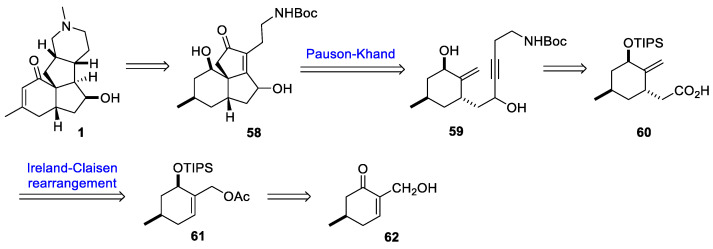
Ishizaki’s 2005 retrosynthesis. TIPS = triisopropylsilyl. Ac = acetate. Boc = *t*-butoxycarbonyl.

In 2005, another report by Ishizaki and coworkers was published describing the formal synthesis of racemic 1 [65]. Equipped with chemistry developed in their lab regarding the intramolecular Pauson–Khand reaction of exocyclic enynes [66,67,68,69,70,71,72], they realized a novel means of constructing the ABC-rings of the magellanine-type diquinane alkaloids. First, the piperidine moiety of 1 was thought to be derived from tricyclic enone 58, whose diquinane 5-5-fused ring system could be constructed from the aforementioned Pauson–Khand reaction of 59 (Scheme 14) [73]. The amino-enyne of 59 can be accessed from carboxylic acid 60, for which the latter can come from the Ireland–Claisen rearrangement of α,ß-unsaturated silyl enol ether 61. Lastly, 61 can be traced back to hydroxy enone 62 [74]. 

**Scheme 15:**
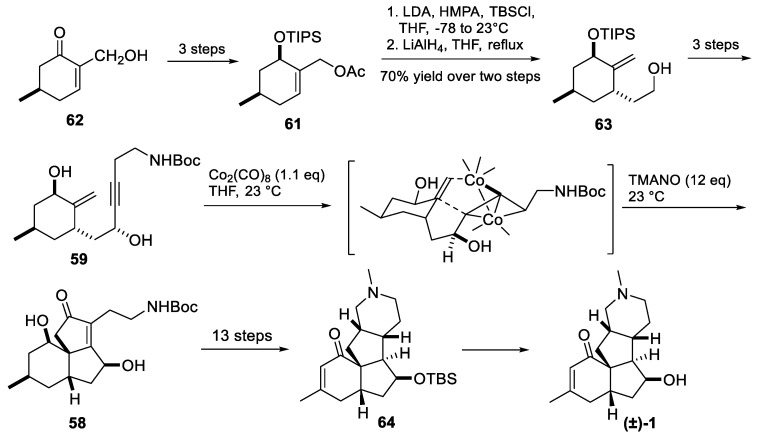
Ishizaki’s 2005 synthesis. LDA = lithium diisopropylamide. HMPA = hexamethylphosphoramide. TBSCl = *t*-butyldimethylsilyl chloride. THF = tetrahydrofuran. TIPS = triisopropylsilyl. TBS = *t*-butyldimethylsilyl. Boc = *t*-butoxycarbonyl. TMANO = trimethylamine N-oxide.

To prepare for their first key transformation, acetate **61** was first synthesized from hydroxy-enone **62** over three steps (Scheme 15). With **61** in hand, the Ireland–Claisen rearrangement occurred with a subsequent LiAlH_4_ reduction [14,74], which furnished primary alcohol **63** at a 70% yield, over two steps. Next, the installation of the alkyl chain was performed, which brought in a latent Boc-protected amine that could be subsequently adjusted, and set up the 1,6-enyne for the Pauson–Khand annulation. After careful consideration of the conditions, enyne **59** underwent Pauson–Khand annulation under cobalt catalysis with a TMANO additive through the transition state that had been shown to furnish tricyclic enone **58** at a 35% yield [75]. From there, the endgame was straightforward: a series of functional group manipulations furnished **64**, which was then transformed to (±)-**1** through Liao’s established condition.

### 3.6. Mukai’s 2007 Synthesis of 1–3

**Scheme 16:**
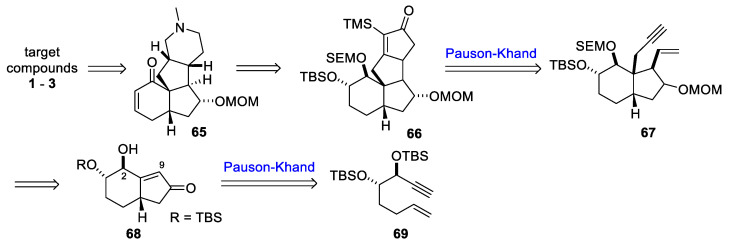
Mukai’s 2007 retrosynthetic analysis. MOM = methoxymethyl. TMS = trimethylsilyl. SEM = trimethylsilyl ethoxymethyl. TBS = *t*-butyldimethylsilyl.

In 2007, the first collective synthesis of **1**–**3** was reported by Mukai and coworkers [76], wherein they detailed a synthetic route to obtain advanced intermediate **65**, from which point a short sequence can be applied to furnish the three target compounds (Scheme 16). Intermediate **65** can be derived from **66** through the expansion of the α-silyl cyclopentenone ring to form piperidine, in addition to introducing unsaturation in the southwestern part of the molecule. Based on work performed in their lab [77,78,79,80,81,82,83,84,85,86,87], intermediate **66** was envisaged to be derived through a cobalt-mediated Pauson–Khand reaction of bicyclic **67**, for which the latter could be constructed from bicyclic enone **68**. The synthesis of **68** would start from another Pauson–Khand reaction of protected 1,2-diol enyne **69**. 

**Scheme 17:**
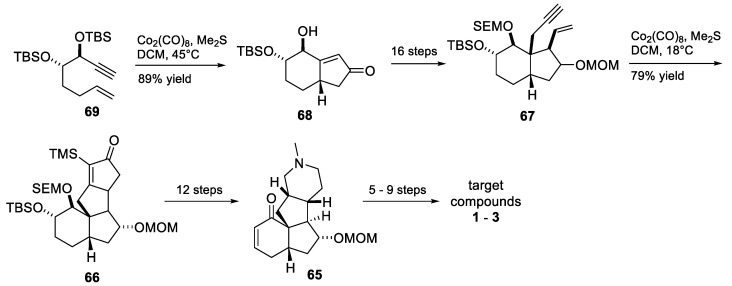
Mukai’s 2007 synthesis. TBS = *t*-butyldimethylsilyl. DCM = dichloromethane. SEM = trimethylsilyl ethoxymethyl. MOM = methoxymethyl. TMS = trimethylsilyl.

To kickstart the desired compound’s synthesis, enyne **69** underwent a Pauson–Khand reaction under cobalt catalysis, which was subsequently mono-deprotected to yield **68** (Scheme 17). Extensive screening efforts surrounding the first Pauson–Khand reaction were undertaken, with Sugihara’s conditions affording **68** with a good yield and stereoselectivity [88]. From there, a 16-step sequence was performed to construct **67** and set the stage for the second Pauson–Khand reaction. This sequence, which is not depicted, started with Ueno–Stork cyclization and subsequent allylation to form a γ-allyl lactone ring [89]. The lactone was then opened to form the diol, while the allyl group was truncated by one carbon to form a vinyl appendage. From there, standard functional group manipulations converted the diol to SEM-protected hydroxy alkyne **67**. With **67** stereoselectively constructed, the second Pauson–Khand reaction took place to construct tetracyclic enone **68** at a 79% yield.

Finally, the elaboration of the enone moiety to a piperidine and the introduction of the C14-C15 unsaturation in 65 were realized through a 12-step sequence. With the advanced common intermediate **65** in hand, a series of five steps furnished **1** and **2,** while a nine-step sequence was required to yield product **3**. 

### 3.7. Yang’s 2014 Synthesis of 1–3

**Scheme 18:**
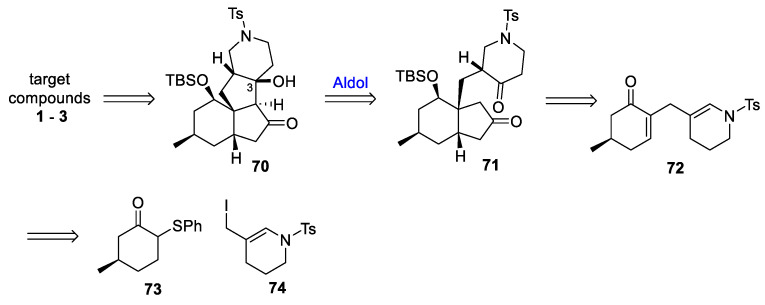
Yang’s retrosynthetic analysis. TBS = *t*-butyldimethylsilyl. Ts = tosyl = *p*-toluenesulfonyl.

In 2014, Yang and coworkers also applied a divergent approach for the synthesis of **1**–**3** from a common intermediate (Scheme 18) [90], but they also recognized the difficulty that their predecessors faced in their respective syntheses in the construction of the D piperidine ring. Thus, they envisioned an ABD to ABCD ring construction approach, where the D ring would be installed at an early stage to circumvent these known issues. Thus, the three target compounds were thought to be traced back to intermediate **70**—itself originating from bicyclic **71**—with a piperidone appendage. The extra hydroxy group at C3 serves an important role in this synthesis. First, it is a labile group that can be removed at any point, but it is ß to a ketone, which allows for a simple intramolecular aldol condensation of diketone **71** [91]. This diketone comes from enone **72** through an annulation, for which the latter can be constructed from alkylation between **73** and **74**. 

**Scheme 19:**
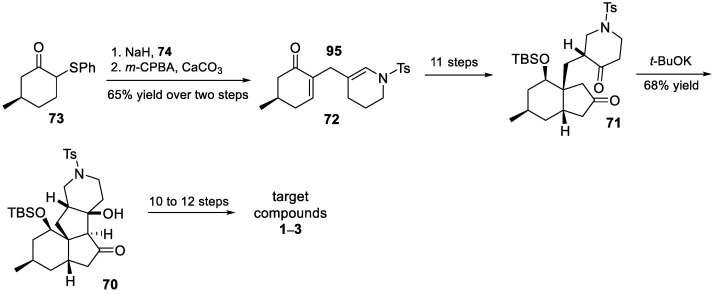
Yang’s 2014 synthesis. *m*-CPBA = *m*-chloroperoxybenzoic acid. Ts = tosyl = *p*-toluenesulfonyl. TBS = *t*-butyldimethylsilyl.

The synthesis commenced with the construction of **72** through the alkylation of **74** onto **73** under NaH conditions, followed by the oxidative elimination of the thiol group through the formation of a sulfoxide with *m*-CPBA (Scheme 19). With **72** in hand, a series of 11 steps were used to install the B ring and set the stage for the intramolecular Aldol addition. First, an allyl chain was installed via conjugate addition of allyl-TMS onto enone **72**, followed by the dihydroxylation of the allyl double bond utilizing osmium tetroxide. From there, a cumbersome four-step sequence was employed to alkylate the product intramolecularly to construct the B ring. This intermediate, not shown, was then oxidized with PCC to furnish diketone **71**, which was suitable for the Aldol addition. For this key transformation, *t*-BuOK was utilized, furnishing **70** at a 68% yield, and thus stereoselectively constructing the tetracyclic core skeleton of these alkaloids. From there, the resulting hydroxy group was removed via Burgess dehydration, whose product was then employed to construct **1** and **2** in ten steps. The endgame synthesis of **3** was interrupted with Mukai’s documented protocol, thereby leading to the completion of the formal synthesis from **74** in 12 steps. Accordingly, Yang and coworkers also synthesized two analogs of (+)-paniculatine, namely 3-hydroxy-13-dehydropaniculatine and 13-*epi*-paniculatine. This report was the first example of constructing the C ring last, which avoided the painful task of stitching the D ring together at a late stage, a sequence many of the previous reports had undertaken. 

### 3.8. Yan’s 2015 Synthesis of 1–3

**Scheme 20:**
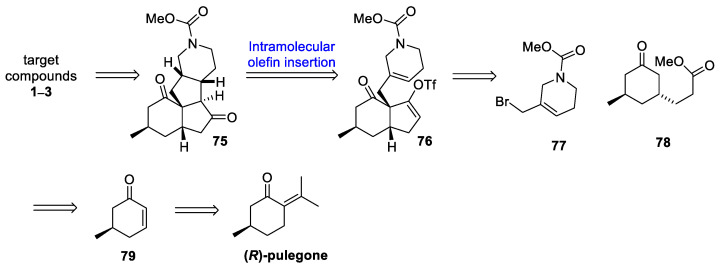
Yan’s retrosynthetic analysis. Tf = triflate = trifluoromethanesulfonate.

Shortly after Yang, Yan and coworkers reported the shortest synthetic strategy (to their knowledge) [92], employing 12–14 steps for the enantioselective synthesis of **1**–**3** (Scheme 20). Their strategy stems from the use of an inexpensive (*R*)-pulegone-derived enone **79** to arrive at the common advanced intermediate **75**. The Pd-catalyzed intramolecular olefin insertion of triflate **76** facilitated the construction of the inner five-membered ring of **75**. The northern tetrahydropyridine could be installed through intermolecular alkylation in tandem with the intramolecular acylation/ring-closure of **78**. The origin of **78** can be traced through standard protocols, in this case employing the use of a Zn-Cu reagent through 1,4-conjugatie addition on enone **79**, which itself can be synthesized from the aforementioned (*R*)-pulegone through known procedures. 

**Scheme 21:**
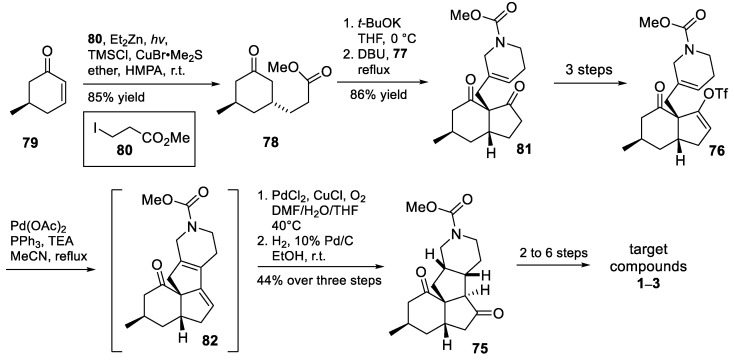
Yan’s 2015 synthesis. TMS = trimethylsilyl. HMPA = hexamethylphosphoramide. THF = tetrahydrofuran. DBU = 1,8-diazabicyclo[5.4.0]undec-7-ene. Tf = triflate = trifluoromethanesulfonate. TEA = triethylamine. DMF = dimethylformamide.

The synthesis leading to their first milestone **78** began with the installation of iodo-alkylester **80** through a copper-catalyzed 1,4-addition of a zinc homoenolate in a complete stereochemical control at an 85% yield (Scheme 21) [93,94]. To construct the B and D rings simultaneously and stereoselectively, tandem base-promoted intramolecular cyclization and intermolecular alkylation were applied. The addition of *t*-BuOK followed by a one-pot addition of DBU and allyl bromide **77** afforded bicycle **81** at an 86% yield. The 5-6-fused diketone ring system forces the active methylene proton into a pseudo-axial conformation, resulting in a top-side attack onto bromide **77**, which results in the R-configuration of the piperidine moiety. With an intramolecular Heck reaction in mind to construct the C ring, a series of three steps was employed to transform diketone **81** into triflate **76**. This was realized through a chemoselective ketalization, conversion of the remaining ketone to its enol triflate, and then the removal of the dioxolane group to furnish **76** at a 73% yield over the three steps. With the stage set, the key Pd-catalyzed intramolecular olefin insertion was performed, exposing **78** to Pd(OAc)_2_, PPh_3_, and Et_3_N, which afforded dienone **82** at an 82% yield. 

With the tetracyclic skeleton constructed, the last challenge of the chemoselective oxidation of the diene was explored. Through the extensive screening of conditions, it was found that an enediketone intermediate (not shown) could be obtained through the treatment of **82** with PdCl_2_/CuCl/O_2_ [95]. This introduced a ketone at C5, while the unsaturation from the piperidine moiety could be removed through hydrogenation with H_2_ and Pd/C. This afforded diketocarbamate **75** at an 86% yield. It was also proven that this three-step sequence could also be performed in one pot, thereby furnishing the desired product with comparable yields to the stepwise approach. 

With the common intermediate **75** in hand, the endgame synthesis of **1**–**3** occurred via a straightforward approach. The selective TBS protection of one of the two ketones, followed by L-Selectride reduction and then deprotection, afforded **1**. For **2** and **3**, L-Selectride was employed first to regioselectively reduce one of the two ketones, after which the protection of the remaining ketone and reduction of the *N*-methylester to its corresponding methyl took place. Lastly, unique to **2** and **3** is the unsaturation present in the western 6-membered ring. This was realized through the addition of *N*-tert-butyl phenylsulfinimidoyl chloride, which dehydrogenated the ketone to its enone. This afforded **2**, which could then be subjected to Dess–Martin oxidation to oxidize the secondary alcohol and thus furnish (+)-magellaninone. 

### 3.9. Qiu’s 2019 Synthesis of (+)-Paniculatine

**Scheme 22:**
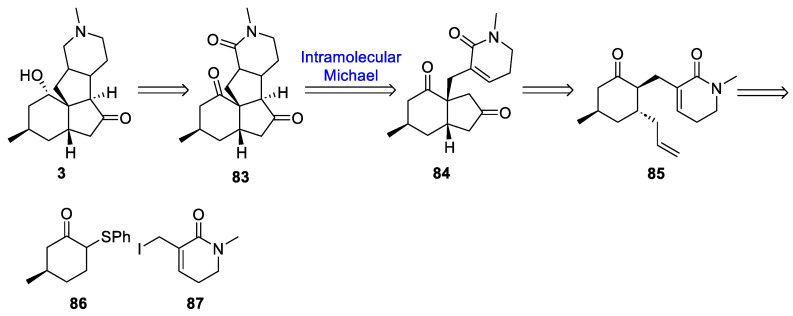
Qiu’s retrosynthetic analysis. TMS = trimethylsilyl.

More recently, Qiu and coworkers reported an asymmetric synthesis of (+)-paniculatine in ten steps from two simple building blocks [96], which, to the best of our knowledge, is the most concise synthesis to date (Scheme 22). They envisaged that **3** could be obtained from the regio- and diastereoselective reduction of tetracyclic triketone **83**. The key transformation in their synthesis was an intramolecular Michael addition in **84** to forge one of the 5-membered diquinane rings in **83**. Bicycle **84** could be obtained from an intramolecular alkylation through an iodohydrin derived from **85**. Lastly, **85** could be stitched together through **86**, **87**, and allyl-TMS. 

**Scheme 23:**
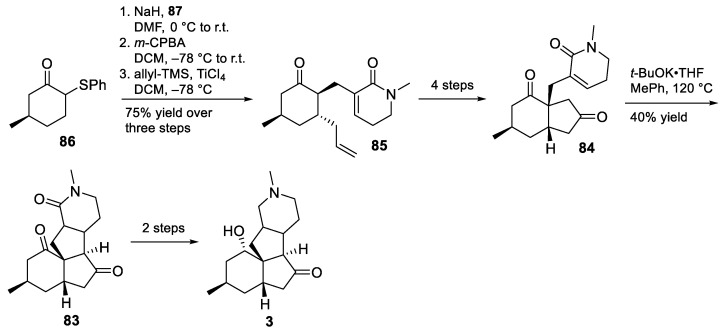
Qiu’s synthesis. DMF = dimethylformamide. *m*-CPBA = *m*-chloroperoxybenzoic acid. DCM = dichloromethane. TMS = trimethylsilyl. THF = tetrahydrofuran.

First, **86** and **87** were subjected to standard alkylation conditions, with the subsequent oxidation of the thioether and elimination of the sulfoxide group, to afford an enone intermediate (not shown). Through Hosomi–Sakurai allylation with allyl-TMS, the side chain was installed, granting **85** at a 75% yield over the three steps (Scheme 23). The diastereoselectivity of the allylation was controlled by the innate stereochemistry of **86**, which is a derivative of enantiopure (*R*)-pulegone. Then, the terminal olefin of **85** was converted to an iodohydrin through NIS [97], followed by intramolecular alkylation to form the five-membered ring. The Dess–Martin oxidation of the secondary alcohol then furnished bicycle **84** at an 84% yield over four steps. While at first the key Michael addition seemed straightforward, this was not the case. This was likely due to the use of an amide as an electron-withdrawing group, which loosely deactivates the α,ß-unsaturated system required for the reaction. Standard bases, such as K_2_CO_3_, DBU, LDA, or NaH, proved ineffective for this transformation. Through significant optimization, they found that *t*-BuOK in dilute toluene under reflux conditions afforded the desired cyclized product **83** at a moderate yield. With the tetracyclic skeleton of paniculatine constructed, two final steps were employed from **83** to complete the synthesis of **3**. First, the protection of the eastern ketone to form TBS enol-ether; then, the L-Selectride reduction of the remaining ketone to the corresponding secondary alcohol; and finally deprotection, which afforded (+)-paniculatine at a 70% yield over the three transformations. 

### 3.10. Yao’s 2022 Synthesis of 1–3

**Scheme 24:**
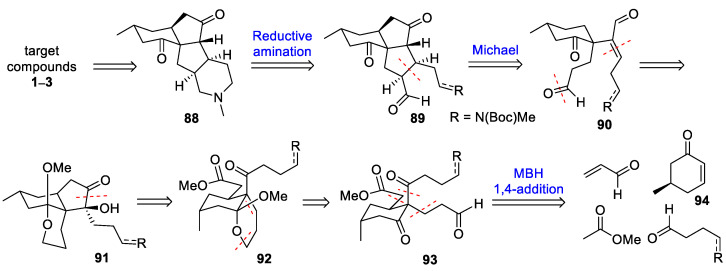
Yao’s retrosynthetic analysis. Boc = *t*-butoxycarbonyl.

To the best of our knowledge, the most recent synthesis was reported in 2022 by Yao and coworkers [98], which consisted of the collective synthesis of **1**–**3** (Scheme 24). They envisioned that the three natural products could be derived from the common intermediate **88**. The piperidine ring of **88** could be derived from the reductive amination of aldehyde **89**, which itself could be obtained from an intramolecular Michael addition of **90**, a common procedure for 1,5-dicarbonyl systems. The enone moiety of **90** could come from the dehydration of tertiary alcohol **91**, for which the latter could come from an intramolecular reductive C-C bond formation between the two carbonyls in **92** [99,100,101]. Methyl ester **92** can result from a functionally decorated intermediate **93**, which, in turn, can be derived from a four-step sequence of the Morita–Baylis–Hillman reaction, alcohol oxidation, and two Michael additions. It is worth noting that Yao and coworkers initially described a synthesis wherein R is a dioxolane acetal instead of a N-Boc group. In their initial synthesis development, they successfully obtained intermediate **89;** however, they decided to revisit their lengthy scheme to 1) shorten the synthesis and 2) introduce the D-ring amine at an early stage. Here, we describe the second edition of their synthesis.

**Scheme 25:**
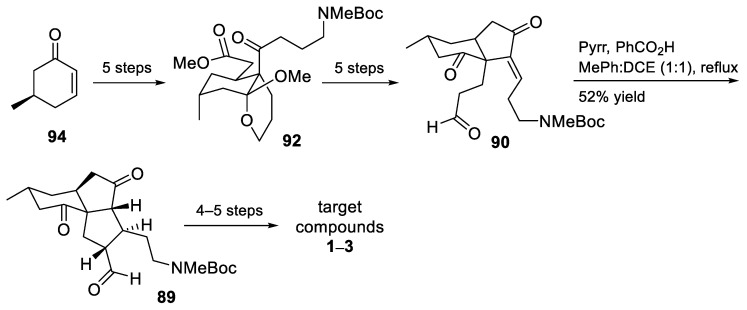
Yao’s synthesis. Boc = *t*-butoxycarbonyl. Pyrr = pyrrolidine. DCE = 1,2-dichloroethane.

The first synthetic goal was the five-step synthesis of chiral intermediate **93** containing a key quaternary carbon center (Scheme 25). First, a Morita–Baylis–Hillman reaction between **94** and the alkyl aldehyde gave a *ß*-hydroxy enone intermediate as an inseparable mixture of diastereomers. The secondary alcohol was then oxidized under Dess–Martin conditions to deliver the 1,3-diketone at a 90% yield. Next, the two Michael additions were implemented: the first to install the enolate derived from methyl acetate and the second with acrolein. This yielded an alkylester-diketone intermediate that was cyclized to form the southern tetrahydropyran ring in **93**. This five-step sequence proved to be a robust procedure that was applied for the large-scale preparation of enantiopure intermediate **93**. With a sufficient quantity of **93** in hand, the intramolecular reductive C-C bond was formed. In their 2012 and 2021 reports for the total synthesis of the fawcettimine-type alkaloids [99,100], Yao and coworkers had discovered that lithium-arenides were capable of forming this type of bond through single-electron reduction. This was applied in the currently discussed route with the use of Li-naphthalide, leading to the production of the resulting tertiary alcohol with high stereoselectivity, which was likely due to the coordinating effects between the lithium ion and the neighboring methoxyl group, which forces the alcohol onto the “top” face. Burgess dehydration then led to the formation of the *α,ß*-unsaturated enone as a Z-isomer, which could be readily converted to an E-isomer **90** through irradiation at 390 nm.

The construction of the C-ring was carried out through the authors key intramolecular cyclization of aldehyde **90**. By analyzing the preceding literature [102,103,104,105,106,107], they had discovered that the use of pyrrolidine and benzoic acid could forge this key C-C bond in order to close the C-ring. Thus, through extensive condition screening, they found that catalytic amounts of pyrrolidine (10 mol%) and benzoic acid (12 mol%) in DCE at reflux or near-reflux conditions produced **89** at moderate to good yield.

Lastly, the endgame synthesis of **1**–**3** was performed through a series of similar steps to the preceding syntheses: the selective reduction of a ketone, the reduction of the remaining ketone, and finally deprotection to furnish the three natural products in an enantioselective fashion.

## 4. Other Notable Syntheses

In this last section, we would like to highlight some of the formal syntheses as well as unique strategies utilized to construct these complex natural alkaloids. This is by no means a comprehensive list; a short scheme of the key transformation will be shown and described. 

**Scheme 26:**
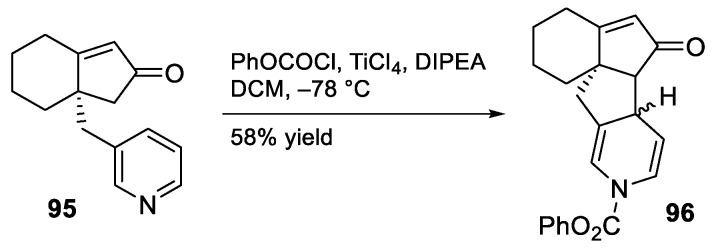
Meyer’s 1995 strategy. DIPEA = diisopropylethylamine. DCM = dichloromethane.

The first strategy to highlight is that reported by Meyers and coworkers in 1995 (Scheme 26) [108]. Their plan hinged on an adaptation of a known regioselective intermolecular 1,4-addition of enolates to activated acyloxypyridinium salts [109,110,111,112,113]. Thus, pyridine **95** was treated with phenyl chloroformate to activate the pyridine ring for nucleophilic attack; meanwhile, the addition of titanium chloride provided the enolate for said attack. The result of this reaction was the 1,4-dihydropyridine product **96** at a 58% isolated yield along with 16% regioisomers. While constituting an incomplete synthesis, this strategy provides an efficient method for constructing all six stereogenic centers contained in magellanine.

**Scheme 27:**
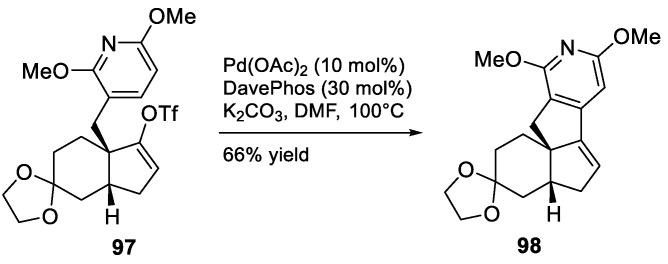
Sarpong’s 2012 strategy. Tf = triflate = trifluoromethane sulfonate. DMF = dimethylformamide.

Some time later, Sarpong and coworkers reported a similar pyridine-derived approach for the construction of the C ring (Scheme 27) [114]. In this strategy, they followed conditions established by Echavarren for the direct regioselective arylation of pyridines with palladium catalysis [115,116]. With the use of Pd(OAc)_2_ (10 mol%) and DavePhos (30 mol%), vinyl triflate **97** was employed to close the C-ring of **98** at a 66% yield. Similar to Meyers, to the best of our knowledge, Sarpong and coworkers have not completed the total synthesis of **1**–**3**; however, another unique approach was disclosed in 2017 [13].

**Scheme 28:**
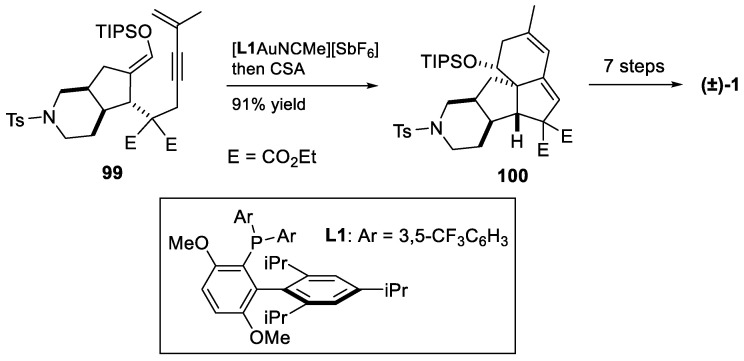
Barriault’s 2017 Strategy. TIPS = triisopropylsilyl. Ts = tosyl = *p*-toluenesulfonate. CSA = camphorsulfonic acid.

Lastly, a more recent strategy was reported by Barriault and coworkers in 2017 (Scheme 28) [117]. Their total synthesis of magellanine was conducted as a result of their lab’s efforts toward the development of a gold(I)-catalyzed dehydro Diels–Alder reaction [118]. In their work, they first identified ligand L1 as optimal for use in conjunction with gold(I) to initiate a stereoselective formal [4+2] between an enyne and an olefin. Thus, enyne **99** was subjected to the shown conditions to afford **100** at a 91% yield. This transformation efficiently constructed the CD-ring in a single step and at an excellent yield and was shown to be sufficiently robust so as to be performed on a gram scale. From there, Barriault and coworkers applied a 7-step synthesis to achieve magellaninone as a mixture of enantiomers over 11 longest linear steps. 

## 5. Conclusions

This review highlights the development of synthetic strategies and their application in the total synthesis of magellanine, magellaninone, and paniculatine, three natural products from the Lycopodium family. Due to the unique 6-5-5-6-fused ring system of these molecules, each author devised a unique and robust method for their careful construction. Of particular note is the construction of the C ring. While groups such as Yang, Qiu, and Yan employed elementary transformations (albeit powerful and robust), the other authors each devised their own unique strategies for the synthesis of this pivotal piece of the puzzle. Another point that we found interesting was that many groups decided to start with a derivative of the A-ring, most often through a pulegone-analog route. From there, each group introduced each of the other three rings in very efficient manners through reliable transformations. We hope that the chemistry introduced in this review may promote future progress in the pursuit of the total synthesis of *Lycopodium* alkaloids and related compounds.
